# From prototype to deployment: An EU-centric lifecycle framework for law enforcement AI

**DOI:** 10.12688/openreseurope.21859.1

**Published:** 2026-04-30

**Authors:** Mikel Aramburu, Jorge García, Seán Gaines, Nizar Touleimat, Ernesto La Mattina, Babak Akhgar, Dimitris Kavallieros

**Affiliations:** 1 Fundación Vicomtech, Basque Research and Technology Alliance, Donosti, Euskadi, 20009, Spain; 2 CEA - List, French Alternative Energies and Atomic Energy Commission Administrative Headquarters, Palaiseau, Île-de-France, 91120, France; 3 AI & DATA R&I Lab - Data Centric AI, Engineering Ingegneria Informatica SpA, Palermo, Sicily, 90146, Italy; 4 Centre of Excellence in Terrorism, Resilience, Intelligence, and Organised Crime Research (CENTRIC), Sheffield Hallam University, Sheffield, South Yorkshire, S1 1WB, UK; 5 Information Technologies Institute (ITI), Centre for Research and Technology-Hellas, Thessaloniki, Makedonia Thraki, 570 01, Greece

**Keywords:** Law Enforcement; Trustworthy AI; MLOps; DevSecOps; EU AI Act; GDPR; AI Governance; Security Research.

## Abstract

Artificial intelligence can substantially enhance law-enforcement capabilities, but its use in security research domains, including Fight Crime Terrorism, Border Management, INFRA, and DRS, raises significant legal, ethical, and operational challenges. Access to operational case data is typically restricted, making it unavailable for continuous ingestion or model training, while the adoption of third-party models, datasets and software artefacts introduces intellectual-property and licensing constraints. At the same time, EU regulations, notably the GDPR, the Law Enforcement Directive (LED), and the EU AI Act, impose procedural and technical safeguards that must be embedded throughout the development lifecycle **. To address these challenges, this paper presents a practical, EU-centric lifecycle framework for developing AI systems in security-sensitive contexts**. The methodology is structured into five stages: Matchmaking, Definition & Design, Development, Validation, and Monitoring. By mapping legal and ethical obligations to concrete engineering checkpoints, the framework supports data provenance, reproducibility, and software supply-chain assurance through artefacts such as dataset registries, Model Cards, and SBOMs. To address restricted access to operational data, the methodology also defines validation patterns for end-user evaluation, including on-premises bring-solution-to-data assessment. The main contributions of the paper are a tailored lifecycle methodology, a compliance mapping linking EU obligations to lifecycle evidence, and a practical assurance package for traceable and auditable development. The methodology is further illustrated through a worked example derived from the STARLIGHT ( https://starlight-h2020.eu/) European project, showing how operational validation can be conducted without exposing raw law-enforcement data.

## Plain Language summary

This paper provides a step-by-step guide for building Artificial Intelligence (AI) systems for security and law enforcement projects in the European Union (EU). Because AI systems in these high-stakes areas must be safe, ethical, and legally compliant, developers must follow strict rules like the EU AI Act and the General Data Protection Regulation (GDPR).

The major challenges in building these systems are that law enforcement agencies cannot easily share highly sensitive, real-world data with developers, the compliance with the legal and ethical European framework and the intellectual-property and licensing constraints. To solve this, our paper proposes a five-stage methodology that allows developers to build, test, and monitor AI models reliably without needing direct access to private operational data and is compliant with both licensing and legal and ethical frameworks. Instead of treating the law as an afterthought or a final checklist, this framework builds legal and ethical checks directly into the daily work of writing code and managing data.

By adopting this approach, technical teams and law enforcement agencies can work together to create AI tools that are effective, trustworthy, and fully aligned with EU regulations, ultimately protecting both the users of the technology and the public.

## Introduction

### Context

Artificial intelligence (AI) is increasingly adopted in security-sensitive domains, where its potential to enhance situational awareness and decision-making must be balanced against legal, ethical and operational constraints. Security-related projects face distinctive challenges that complicate the development of operationally reliable AI and demand a lifecycle approach that embeds compliance, traceability and robustness from the outset.

### Legal and regulatory drivers

The EU regulatory landscape for security and law-enforcement AI combines established data protection requirements with newer, AI-specific lifecycle obligations. GDPR ^1^ and the Law Enforcement Directive (LED) ^2^ regulate the handling of personal data in operational contexts, while the EU AI Act ^3^ introduces a risk-based framework that, particularly for high-risk uses, requires evidence of safety, accountability and governance across the system lifecycle. In practice, these instruments make compliance a continuous engineering requirement, strengthening the need for auditable evidence (e.g., traceability, documentation and monitoring outputs) that can be produced and maintained throughout development and deployment.

### Key challenges

Despite advances in tooling and practice, we did not identify in the literature a single, widely accepted procedure for developing AI systems tailored to security contexts. The main obstacles are:

• **Data-related challenges**. Operational data held by law enforcement agencies is often legally and procedurally restricted, limiting access to representative case data. Developers therefore rely on public benchmarks, synthetic or generative samples, or purpose-built data collection campaigns. In parallel, provenance and licensing constraints restrict the reuse and distribution of training data and model components.
• **Model- and lifecycle-related challenges**. Contemporary MLOps practices ^4, 5^ commonly assume continuous ingestion of production data and rapid retraining cycles, an operational model that may be infeasible when live law-enforcement streams cannot be shared with developers or used for continuous improvement. Moreover, the adoption of third-party models, pretrained weights and libraries introduce IP, licensing, and usage-restriction constraints that can directly affect deployment.
• **Legal, ethical and transparency challenges**. EU obligations require that legal and ethical safeguards be engineered into the lifecycle through structured processes and evidence. This includes risk and impact assessments (e.g., DPIAs ^6^), traceability of data and training procedures, transparency documentation and post-deployment monitoring. These requirements, if treated as afterthoughts, are difficult to satisfy credibly in high-risk contexts.

These challenges are further amplified by the diversity of stakeholders involved in EU security ecosystems (practitioners, researchers, industry, civil society, policy makers and agencies), whose expectations and constraints must be reconciled within a single development and governance process.

### Contribution

This paper addresses the development of AI-based solutions for security and law-enforcement applications in the European Union (EU), where data sensitivity, regulatory scrutiny and operational risk are particularly acute. In such settings, deploying AI requires more than model accuracy: it demands a lifecycle approach that supports traceability, reproducibility, legal and ethical compliance, and operational robustness. Motivated by experience in security-oriented projects and by evolving EU obligations (particularly the AI Act ^3^ and GDPR ^1^), we propose a practical lifecycle methodology intended to bridge the gap between laboratory-tested research components and deployable, auditable systems.

We present a five-stage lifecycle methodology: *Matchmaking*, *Definition & Design, Development, Validation* and *Monitoring*, which are described in the *Security-oriented AI lifecycle framework* and subsequent stage-specific sections. The methodology is technology-agnostic at its core and is organised around governance gates, responsibilities and evidence artefacts that enable continuous assurance. It explicitly targets contexts where operational law-enforcement data cannot be directly ingested into standard development loops, and it therefore prescribes alternative data strategies, provenance practices, and end-user validation patterns under operational constraints, particularly in the *Validation* and *Monitoring.*

The main contributions are:

1. **An operational lifecycle framework for security and law enforcement AI projects.** An end-to-end development lifecycle tailored to constrained-data security contexts, specifying stages, governance gates and evidence requirements that align engineering practice with operational constraints.
2. **A preliminary EU regulation-centric compliance and governance mapping.** A mapping of EU obligations (AI Act, GDPR/LED and related instruments) to lifecycle artefacts and decision gates (e.g., risk management records, DPIAs, traceability documentation and transparency artefacts) so legal duties are embedded into the development process (outlined through Section 3).
3. **A practical assurance checklist of engineering patterns and artefacts**. A checklist-oriented set of recommended practices that can be integrated into development pipelines, including reproducibility controls, provenance tracking, license/IP considerations, software supply-chain measures (e.g., SBOMs ^7^), and post-deployment monitoring practices (outlined across Section 3 and provided as a consolidated checklist in the Supplementary Materials).

The remainder of the paper first surveys related work on EU trustworthiness requirements and AI lifecycle engineering in security-sensitive settings, as well as presenting some of the projects and initiatives that influenced this work. It then presents the proposed lifecycle methodology (Matchmaking, Definition & Design, Development, Validation and Monitoring), describing for each stage the provider–deployer responsibilities, governance gates, and the engineering artefacts used to support traceability and auditability. A compliance-mapping section links core EU obligations to lifecycle evidence. The paper concludes with recommended validation patterns for constrained-data environments, post-deployment monitoring and feedback loops, and a discussion of remaining challenges for deploying trustworthy AI in the European security domain. To illustrate the practical application of the methodology in the *Illustrative application of the lifecycle methodology* ^8^ presents a worked example derived from the STARLIGHT European project, ^9^ showing how the lifecycle can be applied under real operational constraints.

## Previous work

The development of AI systems for Law Enforcement Agencies (LEAs) and security applications sits at the intersection of strict EU governance requirements, rapidly evolving engineering practices (MLOps/DevSecOps), and operational constraints such as restricted data access and isolated infrastructure. While each of these strands is well covered in the literature, they are rarely synthesised into an integrated lifecycle tailored to high-risk, constrained-data security settings.

### The regulatory landscape: From abstract principles to engineering practice

The European Union (EU) has promoted “trustworthy AI” as a combination of lawfulness, ethical safeguards and technical robustness. ^10^ The EU AI Act operationalises this agenda through a risk-based regime that, in many law-enforcement and security use cases, entails stringent lifecycle obligations, including risk management, documentation, traceability, transparency and post-deployment monitoring. ^11, 12^ Prior work highlights a persistent translation gap between legal mandates and day-to-day engineering decisions: requirements are often handled as external checklists rather than embedded as design constraints throughout the lifecycle. ^13, 14^ Recent contributions propose compliance-oriented documentation templates assessment tools, and machine-readable compliance frameworks that structure obligations across lifecycle phases and requirement categories. ^12, 15, 16^ However, these efforts primarily emphasise formalisation, reporting or compliance modelling rather than guiding engineering workflows end to end.

### Lifecycle methodologies: Fragmentation of security and compliance

To manage complexity and accelerate delivery, MLOps, DevOps and DevSecOps promote automation and standardisation across the AI lifecycle, including continuous integration, testing, deployment and monitoring. ^17, 18^ Emerging proposals such as compliance-aware pipelines and audit-oriented tooling attempt to align DevOps velocity with governance requirements by strengthening traceability and audit trails within delivery pipelines. ^19, 20^ However, several studies note that security and compliance are still frequently treated as parallel activities that are “bolted on” to pipelines rather than used as drivers that shape requirements, data strategies, validation protocols and monitoring from the outset. ^21, 22^ Moreover, robustness is commonly operationalised as technical stability (e.g., resilience to drift), which does not fully capture the procedural accountability and transparency expectations associated with high-stakes security deployments.

### The gap: constrained-data security environments

The integration problem is most acute in constrained-data environments typical of LEA and security applications, where operational data may be scarce, sensitive, classified or legally inaccessible to developers, and where infrastructure constraints (including isolated or air-gapped deployments) limit common MLOps assumptions. ^23^ Although the literature offers partial solutions, privacy-preserving learning methods, scarcity mitigation, governance templates, compliance modelling and pipeline automation, ^16– 18, 24^ it lacks a unified EU-compliance-to-engineering lifecycle that provides practical guidance on building auditable, deployable systems under restricted data access and strict accountability requirements. ^12, 25, 26^

In summary, while the “what” (EU governance requirements) and the “how” (MLOps/DevSecOps automation) are individually well documented, the literature still lacks a practical, integrated framework for constrained-data law-enforcement and security AI that simultaneously addresses:

1. **EU high-risk compliance as an engineering output**: How to produce and maintain auditable lifecycle evidence (risk management, documentation, traceability, monitoring) rather than treating compliance as a post hoc checklist.
2. **Engineering robustness with security-by-design**: How to embed security controls and continuous assurance (testing, supply-chain controls, vulnerability/licence checks, secure deployment practices) directly into AI delivery pipelines, instead of running them in parallel.
3. **Operational data constraints**: How to develop, validate and monitor systems when operational data is scarce, sensitive, or inaccessible to developers (including isolated/air-gapped deployments), while still meeting performance and accountability expectations.

## Methodological foundations from EU initiatives

The methodology presented in this paper synthesises practical experience from security-oriented projects, current state-of-the-art AI development practices, and expert knowledge. It is intended as a pragmatic guide for security-focused AI development, promoting compliance with EU regulations and transparency in system behaviour for end-users.

Rather than building a framework from scratch, this methodology integrates and operationalises the outputs of several major EU-oriented initiatives that promote explainable, maintainable, and transparent AI systems. Table 1 summarises these foundational projects and illustrates exactly where their tools and conceptual frameworks have been integrated into our proposed lifecycle stages.

**Table 1.** Methodological foundations from EU projects and initiatives.

EU Project/Initiative	Core Contribution & Focus	Integration into Proposed Methodology
STARLIGHT ^9^	**Agile Co-Creation**: Short, targeted mini projects where technical developers and end users collaborate iteratively to adapt AI tools to specific operational needs.	Maps directly to the organisational cadence of the **Matchmaking, Definition & Design, Development, and Validation** stages, ensuring continuous LEA engagement.
LAGO ^27^	**Trusted Research Data Ecosystem**: A shared research data space and suite of tools supporting the data lifecycle, including anonymisation, synthetic data generation, and secure publishing.	Operationalises the **Data Processing & Governance** pipeline within the Development phase, mitigating data access barriers while preserving privacy.
AP4AI ^28^	**Accountability Assessment**: A framework assessing systems across design, data, legal compliance, and oversight, explicitly mapped to the EU AI Act’s obligations (e.g., Risk management, FRIA).	Embedded as the primary evaluation mechanism during the **Legal and Ethical Pre-assessment ** (Definition & Design) and to verify compliance coverage throughout the lifecycle.
MultiRATE ^29^	**Holistic Readiness Levels (RLs)**: A unified scale aggregating Technology, Societal, Security, Legal, Privacy and Ethics, Integration, Commercialisation, Manufacturing, and a Holistic RL ^30^ readiness into a single evaluation method.	Acts as a cross-cutting control across all **Governance Gates**, informing go/no-go decisions from Definition & Design through Monitoring.
ALTAI ^31^	**Trustworthy AI Checklist**: A principle-based self-assessment mapping criteria like human agency, robustness, and transparency to actionable checks.	Utilised as a repeatable checklist from **Pre-assessment ** through **Validation**, translating abstract principles into concrete artefacts like DPIAs and Model Cards.

## Security-oriented AI lifecycle framework

The methodology section proposes a security-oriented AI lifecycle framework tailored to law enforcement and security deployments. It outlines the actions and processes used to develop an AI-based solution within a security-oriented AI organisation, and it shows how the developer and the end user interact at each stage of the project. The framework specifies an end-to-end workflow that structures collaboration between technical providers (often involving R&D institutions, private external companies, or different departments within the same organisation that do not ordinarily collaborate) and operational deployers (LEAs). The methodology translates regulatory and operational constraints into concrete governance gates and evidence artefacts.

**Figure 1.**
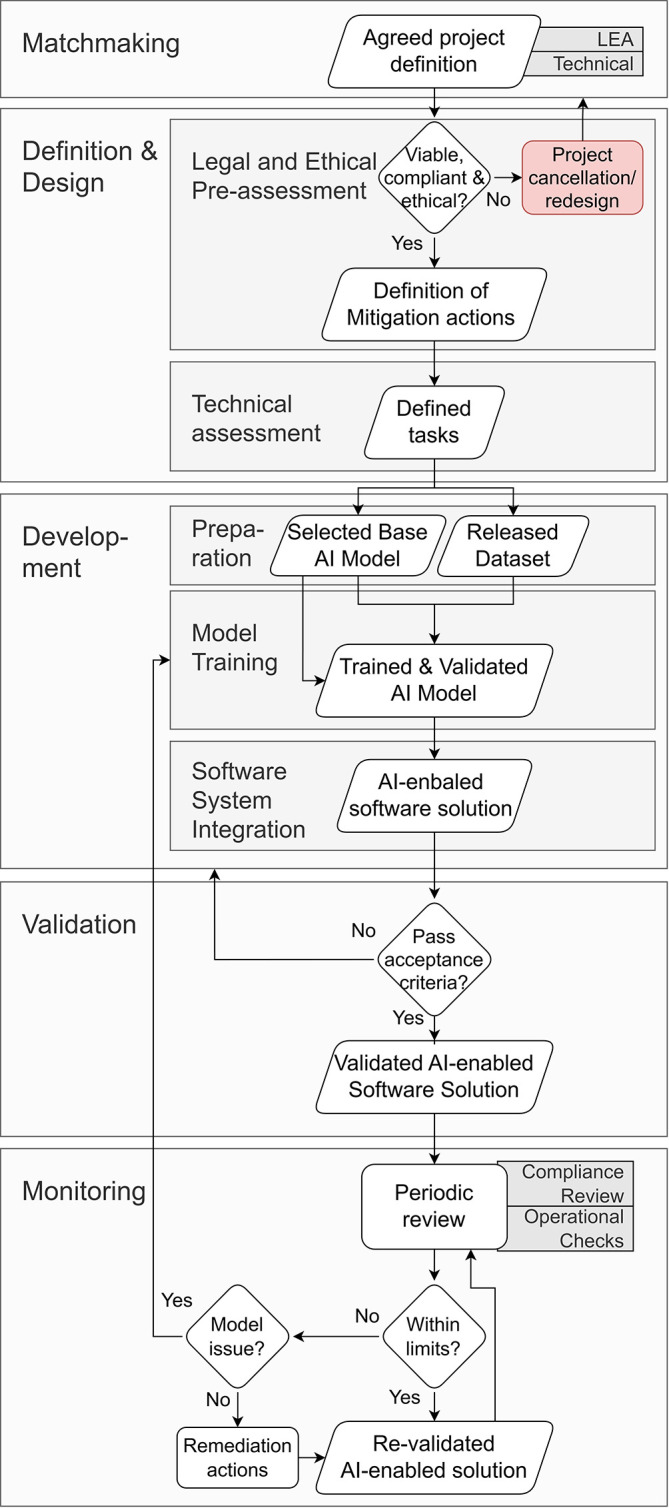
Overview of the proposed lifecycle methodology for developing AI-enabled solutions in security-sensitive environments.

The lifecycle proposed in this paper is structured around five principal steps, as drawn in Figure 1: **Matchmaking**, where operational needs, constraints and expectations are aligned and an initial feasibility and risk screening is performed; **Definition & Design**, during which use cases are formalised, functional and non-functional requirements are fixed, and technical, legal and ethical boundaries are translated into concrete specifications; **Development**, which covers dataset curation, model engineering, versioning, CI/CD and deployment pipelines so the AI component becomes an integrated software product; **Validation**, where the delivered solution is tested, evaluated against agreed metrics, audited for regulatory and ethical compliance, and iterated on with end-user feedback until it meets operational acceptance criteria; and **Monitoring**, where the solution is periodically reviewed and re-validated in legal and ethical, security and technical performance aspects.

To support interpretability of the framework, the *Illustrative application of the lifecycle methodology* ^8^ provides a worked example derived from the STARLIGHT project, illustrating how the proposed lifecycle stages and governance gates were applied in a real security-oriented AI deployment.

### Structure of the lifecycle stages

To support the proposed methodology, which is both standardised and highly actionable, each of the five lifecycle stages is detailed using a consistent, six-part framework. This structure explicitly bridges the gap between operational constraints and engineering execution:

• **Challenge**: Identifies the specific legal, operational, or data-related constraints (e.g., lack of access to raw law enforcement data, third-party licensing risks) that make this stage uniquely difficult in security contexts.
• **Objective**: Defines the primary goal of the stage and what it aims to achieve to move the system closer to a trustworthy deployment.
• **Operational Approach**: Details the practical and methodological mechanisms used to achieve the objective, explaining *how* the step is executed under restricted conditions.
• **Key Activities**: Provides a concise, actionable checklist of the core engineering, organisational, and legal tasks required during the phase.
• **Governance Gates and Evidence**: Specifies the formal “go/no-go” decision criteria necessary to pass to the next stage, along with the concrete, auditable artefacts (e.g., Model Cards, SBOMs, DPIAs) that must be produced as proof of compliance.
• **Primary Responsibility**: Delineates the ownership of the stage, clarifying whether the **Provider** (R&D institution, private external company, or a different department), the **Deployer** (Law Enforcement Agency), or both are responsible for execution and sign-off.

### Matchmaking

**Challenge.** Synchronising the Deployer’s operational needs with the Provider’s technological capabilities. Because these stakeholders operate in distinct professional domains with different vocabularies, bridging this semantic gap is critical. The primary difficulty lies in reaching a mutually satisfactory agreement where both parties share a fully aligned, unambiguous understanding of exactly what will be developed. Bridging this linguistic divide to successfully synchronise requirements, technologies, and project vocabulary is a critical first step.

**Objective.** The alignment among all stakeholders. Once this mutual understanding is achieved, the phase facilitates a structured convergence to determine if the operational demands and technical feasibility can be successfully aligned under project constraints before formal system design begins.

**Figure 2.**
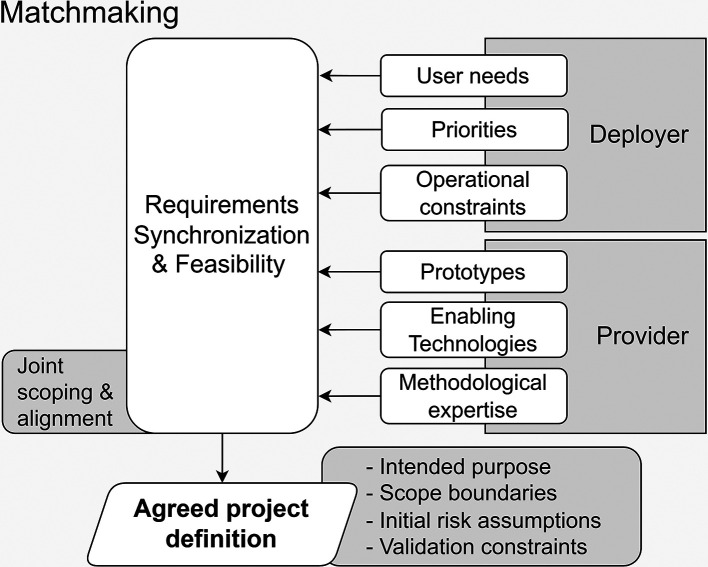
Matchmaking alignment of Deployer operational needs with Provider technical capabilities.

**Operational approach.** This stage functions as a structured pre-design assessment aimed at bridging the semantic gap between stakeholders, rather than serving as a mere informal project scoping exercise. It centres on a joint feasibility study to synchronise the asymmetric inputs from both sides, as shown in Figure 2.

To reach a mutually satisfactory agreement and an unambiguous understanding of the system to be developed, Deployers (LEAs) and technical Providers must reconcile their distinct domain expertise. Table 2 summarises the contribution of the main stakeholders.

**Table 2.** Deployers and Providers contributions.

Deployers contribute	Providers contribute
Operational objectives and user needs	Existing research results and prototypes
User priorities and real-world operational gaps	Available enabling technologies
Context-specific operational constraints	Methodological expertise

Because these stakeholders operate in distinct professional domains, these inputs are inherently asymmetric. Providers typically cannot access live operational data to assess feasibility, while Deployers may lack detailed knowledge of algorithmic limitations. Furthermore, regulatory classifications (e.g., potential high-risk categorisation under the EU AI Act) dictate the design space from the outset. Therefore, the operational approach must actively translate these asymmetrical inputs into a unified, shared conceptual framework.

**Key Activities.**

• Semantic alignment: Establishment of a shared project vocabulary to bridge domain-specific gaps and provide all stakeholders with an identical understanding of system concepts and limitations.
• Requirements synchronisation: Joint articulation of the Deployer’s operational needs mapped directly against the Provider’s technical capabilities to define a realistic, agreed-upon intended purpose.
• Risk screening: Early identification of fundamental rights implications and operational risks based on the proposed technological approach.
• Accountability mapping: Definition of preliminary Provider–Deployer role allocation to support mutual satisfaction regarding project responsibilities.

**Governance Gate and Evidence.** The stage concludes with a formal decision to proceed to the next phase. If conceptual alignment and technical feasibility cannot be mutually synchronised, the use case must be reformulated or discontinued. To pass this gate, specific conditions must be met and backed by auditable evidence artefacts as showed in Table 3.

**Table 3.** Governance gates and required artefacts for Matchmaking.

Governance Gate	Required Evidence/Artefact
The intended purpose is clearly defined and unambiguously understood by both parties.	Documented intended purpose and scope.
Early operational risks and regulatory implications (including high-risk categorisation) are acknowledged.	Preliminary risk and rights-impact screening.
Provider and Deployer responsibilities are provisionally allocated.	Initial role allocation statement.
Final Approval.	Decision record to proceed to Definition & Design.

**Primary Responsibility.** This stage is co-owned by the Deployer and the Provider. The Deployer validates operational necessity and legal permissibility. The Provider evaluates technical feasibility, methodological constraints and their technological capacity to fulfil the Deployer’s needs.

### Definition & Design

**Challenge.** The main challenge of this stage is to establish, before implementation begins, whether the proposed project is feasible and permissible under the current EU legal and ethical framework. In security-sensitive and law-enforcement contexts, feasibility extends beyond technical viability: it depends on regulatory permissibility, lawful personal-data processing under GDPR/LED, compliance with applicable AI Act obligations, and ethical acceptability in the intended operational setting. The core difficulty of this stage, therefore, lies in the legal and ethical pre-assessment, through which Provider and Deployer jointly determine whether the project can proceed, must be redesigned, or should be discontinued.

**Objective.** To translate the agreed project definition into a formally specified AI system concept that is legally grounded, operationally bounded, and technically structured. In constrained-data security environments, this stage is critical because regulatory applicability, data governance limitations, and accountability requirements must be embedded into the system concept before implementation begins. The objective is therefore not only to design a technical solution, but to define the conditions under which it may be lawfully, safely and responsibly deployed.

**Figure 3.**
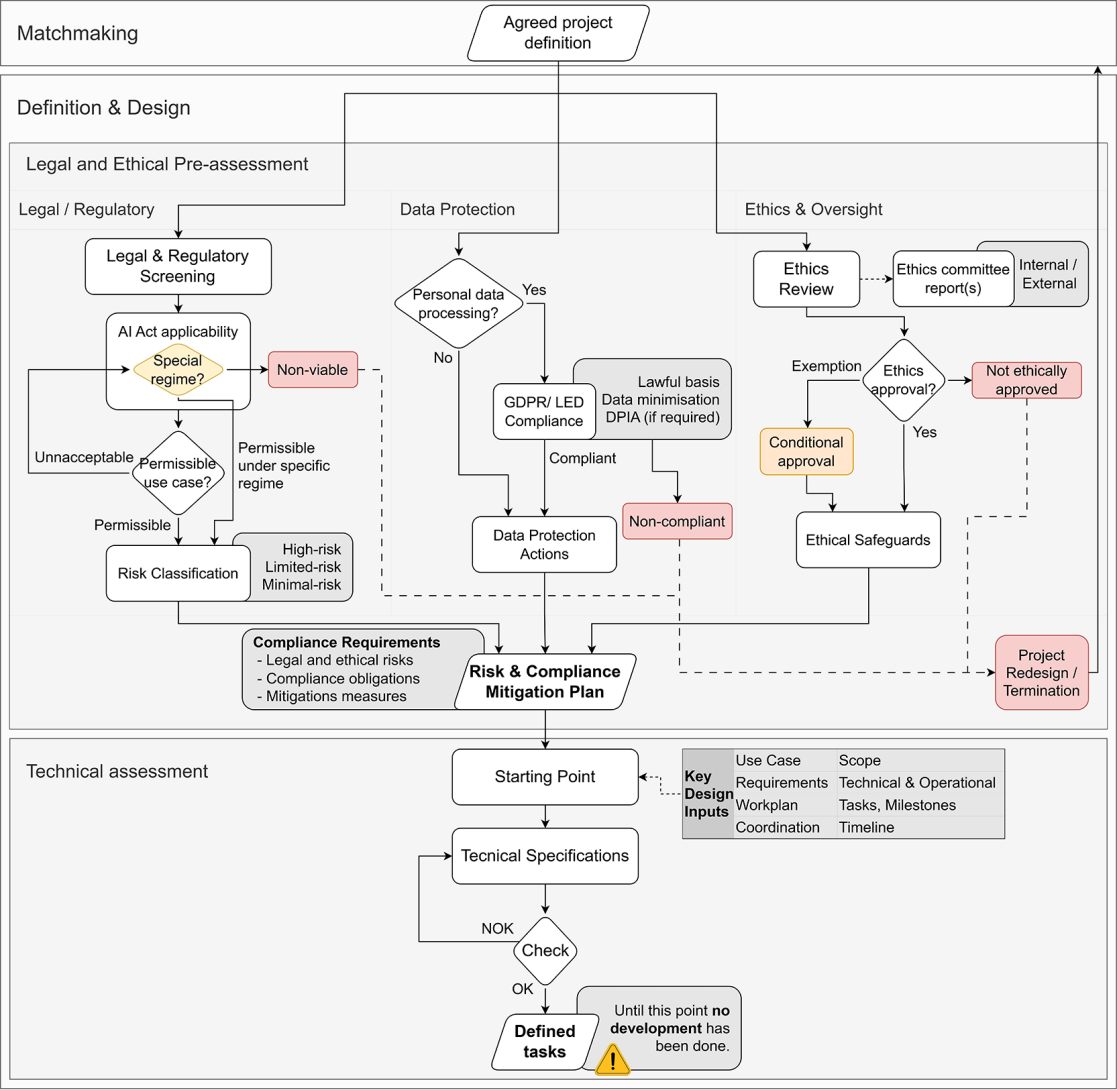
Definition & Design stage with legal, data protection, and ethics pre-assessment.

**Operational Approach.** As illustrated in Figure 3, the Definition & Design stage combines two coupled tracks:

(i) A legal and ethical pre-assessment, and
(ii) A technical assessment.

The legal and ethical pre-assessment is organised around three complementary governance lanes [1]:

• Legal/Regulatory, which evaluates regulatory permissibility, AI Act applicability, special regimes where relevant, and the resulting risk classification.
• Data Protection, which determines whether personal data is processed and, where applicable, applies GDPR/LED compliance conditions such as lawful basis, data minimisation, and DPIA requirements.
• Ethics & Oversight, which evaluates the project through ethics review, committee reporting, and approval or conditional approval mechanisms.

The outputs of these lanes are consolidated into compliance requirements and a risk and compliance mitigation plan, which together define the conditions for subsequent technical design and development. If any governance lane fails, the project must be redesigned or discontinued before progressing further.

The ethics and oversight lane may function as a project-level go/no-go mechanism, since ethics committees or equivalent institutional bodies can approve, condition, or block progression depending on the proportionality and societal implications of the proposed use case.

**Key Activities.** Unlike conventional design phases, these activities explicitly treat regulatory and accountability requirements as design constraints rather than external review criteria.

• Legal and ethical pre-assessment (governance track)
  ○ Define the intended purpose, operational context, and system boundaries, including explicit limitations and non-goals.
  ○ Assess regulatory permissibility and AI Act applicability, including whether the use case falls under a special regime or requires additional constraints.
  ○ Determine the initial risk classification and identify legal, ethical, and fundamental-rights risks.
  ○ Assess whether personal data processing is involved and, if so, define the applicable GDPR/LED compliance conditions.
  ○ Conduct ethics review, including internal and/or external committee consultation where required.
  ○ Identify mitigation actions, conditional requirements, and redesign needs where governance conditions are not fully satisfied.
• Technical assessment (engineering track)
  ○ Define functional and non-functional requirements, including performance, robustness and operational constraints.
  ○ Specify software validation requirements in a User Acceptance Testing (UAT) protocol (defining test scenarios, executing tests based on real-world usage, documenting results, and ensuring that any issues are addressed).
  ○ Specify the system at a conceptual architecture level (components, interfaces, data flows).
  ○ Define the data strategy and governance controls consistent with access restrictions.
  ○ Define the human oversight concept in operational terms (roles, escalation pathways, intervention points).
  ○ Define the validation plan outline (metrics, scenarios, acceptance thresholds, and test conditions).

**Governance Gate and Evidence.** The Definition & Design stage concludes with a formal design approval gate that authorises progression to Development. If these conditions cannot be met, the project is redesigned or discontinued before development starts. To pass this gate, specific conditions, specified in Table 4, must be verified against auditable evidence artefacts.

**Table 4.** Governance gates and required artefacts for Definition & Design.

Governance gate	Required Evidence/Artefact
The intended purpose and system boundaries are documented, including limitations and non-goals.	Intended purpose and system boundary specification, including limitations and prohibited uses.
Initial legal, ethical, and operational risks have been identified, and a mitigation approach is defined.	Pre-assessment record and risk & compliance mitigation plan.
Regulatory applicability screening is completed, and any special regime or design constraint is documented.	Regulatory applicability screening record and risk classification record.
Personal data implications are assessed and, where relevant, GDPR/LED compliance conditions are defined.	Data protection compliance record, including lawful basis, data minimisation measures, and DPIA requirement status.
Ethics review is completed, and the project is approved, approved with conditions, or redirected for redesign.	Ethics review record, ethics committee report(s), and conditional approval record, where applicable.
The data strategy is feasible under operational constraints and includes governance controls.	Data governance and sourcing strategy.
Oversight responsibilities and validation criteria are defined before implementation.	Conceptual architecture, oversight concept/responsibility allocation, and validation plan outline with acceptance criteria.

**Primary Responsibility.** This stage remains collaborative but shifts toward Provider-led system specification, informed and validated by the Deployer. The Provider formalises the technical and methodological design and consolidates the evidence artefacts. The Deployer contributes operational constraints, confirms feasibility within institutional governance, and validates that the specified intended purpose, data strategy, oversight concept, and acceptance criteria align with operational reality, legal obligations, and accountability requirements.

### Development

The Development stage implements the system defined in the Definition & Design phase under controlled, traceable and reproducible conditions. In security and law-enforcement contexts, development is not merely model construction; it is the operationalisation of design constraints, risk mitigation measures and governance requirements defined earlier. All implementation activities must remain consistent with the intended purpose, system boundaries, and regulatory applicability established during the pre-development gate.

**Figure 4.**
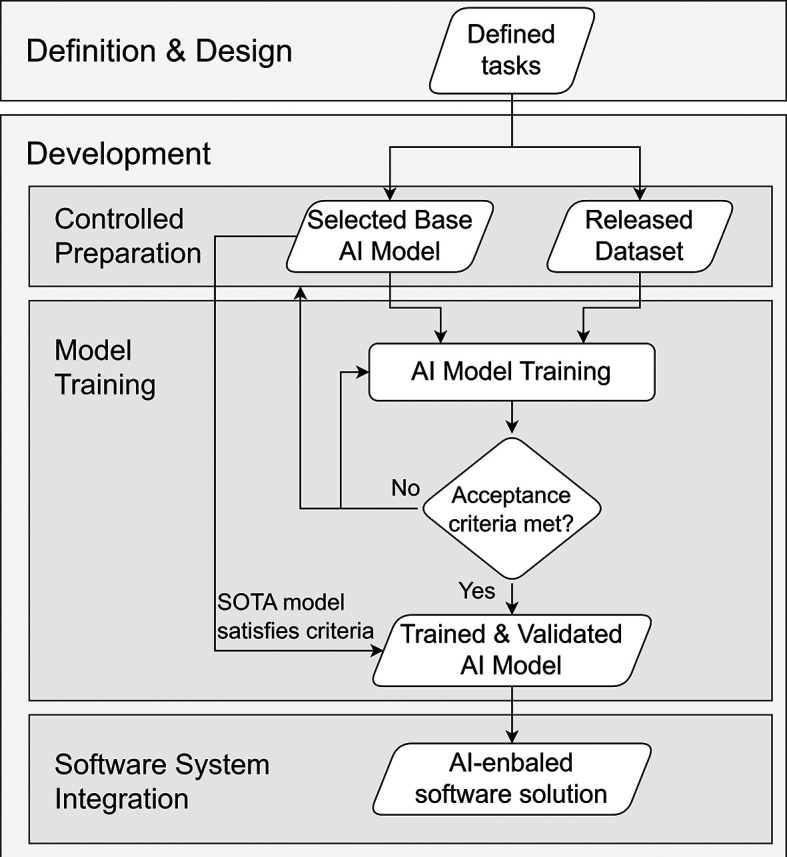
Development workflow for AI-enabled solutions.

To achieve a legally and technically compliant implementation under these constraints, development is therefore structured into four coordinated components (see Figure 4):

(i) Base AI Model selection or construction,
(ii) Data Processing & Governance implementation,
(iii) Model Training and validation, and
(iv) Software System Integration of an AI-enabled solution.

**Cross-cutting constraints: Licences and IP management**

Throughout development, intellectual property rights and licences must be rigorously managed when selecting model architectures, datasets, and third-party software libraries. Licences dictate permitted uses, modification rights, and redistribution rules, carrying severe legal and operational consequences for security projects. For instance, while permissive licences generally suit confidential LEA deployments, strong copyleft or policy-driven licences (e.g., Open RAIL) may impose redistribution obligations or explicitly prohibit law-enforcement use. Table 5 summarises these implications.

**Table 5.** Summary of software licence types and their implications for security-oriented projects.

Type	Example Licences	Key Characteristics	Implications for Security-oriented projects
Permissive	MIT, ^32^ Apache 2.0, ^33^ BSD ^34^	Minimal restrictions. Free use, modification, distribution. Attribution is usually required.	Low risk. Widely compatible with LEA confidentiality and operational use.
Weak Copyleft	MPL 2.0, ^35^ EPL, ^36^ LGPL ^37^	Modifications to covered files must be shared, but larger works may remain proprietary.	Medium risk. Safe if used internally; redistribution obligations may apply.
Strong Copyleft	GPL, ^38^ AGPL ^39^	Derivative works and services must publish source code under the same licence.	High risk. Conflicts with the confidentiality and security needs of LEAs.
Responsible AI Licences	CreativeML, ^40^ Open RAIL variants (Open RAIL-M, Open RAIL++) ^41^	Permissive use but with restrictions on harmful or unethical applications (e.g. surveillance, disinformation).	Variable risk. LEAs must verify that operational uses do not fall under prohibited categories.
Restrictive/Proprietary	Commercial proprietary, ^42^ Research/Custom licences ^43^	Vendor-controlled or limited to academic/non-commercial use. No modifications unless allowed.	Medium to high risk. Often costly, risk of lock-in, and sometimes unusable without renegotiation.

***Base AI Model***

**Challenge.** The main challenge in this component is to select a base model that is both technically suitable and deployable under the project’s legal, operational, and ethical constraints. Although the reuse of pretrained models and research prototypes is common, third-party models may introduce licensing and intellectual property restrictions that can limit or even prevent lawful deployment by the deployer. In addition, the selected model may carry inherited limitations, including bias, domain mismatch, or undocumented assumptions from prior training. A further challenge is to avoid choosing overly general models whose capabilities or behaviour extend beyond the intended use case, thereby increasing governance, validation, and oversight risks.

**Objective.** The Base AI Model component determines the foundational model architecture or pre-existing model artefact on which the solution will be built. This may involve selecting an existing pretrained model, adapting prior research prototypes, or designing a new architecture when existing options are unsuitable for the intended purpose, deployment constraints, or compliance requirements.

**Figure 5.**
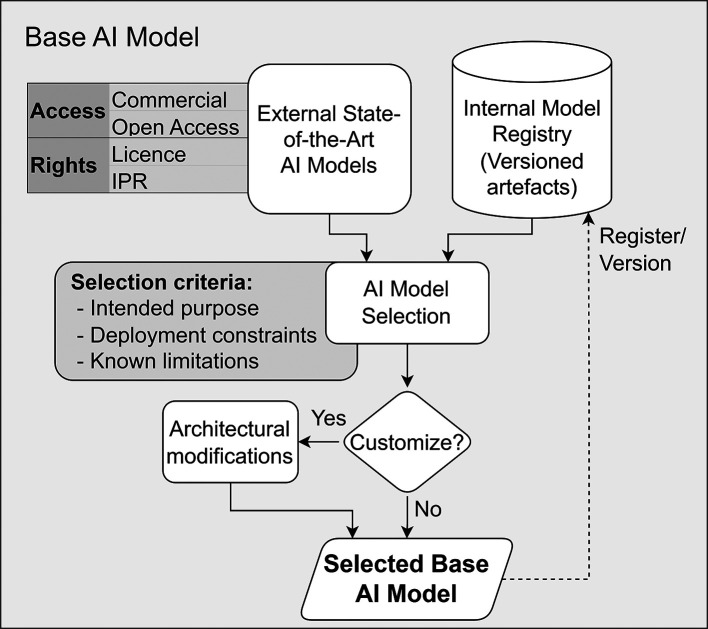
Base AI model selection under access and rights constraints.

**Operational Approach.** As illustrated in Figure 5, candidate base models typically originate from two sources: **external state-of-the-art releases and internal model repositories or registries** maintained by the technical partner. In these collaborations, reuse of pretrained models and research artefacts is common; however, selection must be treated as a governed engineering decision rather than an informal choice. The selection process, therefore, includes an explicit decision on whether the chosen base model can be used “as-is” or requires adaptation (i.e., architectural modifications to the model), with all outcomes being registered and versioned to support traceability.

**Key activities**

• Assess alignment with the **intended purpose** and operational constraints defined in the pre-development gate.
• Evaluate compatibility with data-access limitations and infrastructure constraints (including restricted or isolated environments).
• Review **Licensing and IPR conditions** associated with third-party models, including usage restrictions that may affect the ability to deploy.
• Identify known **limitations**, bias risks, or domain gaps relevant to the deployment context.
• Feasibility of training, inference and maintenance given computational and operational constraints.

**Governance Gate and evidence.** The selection of the base model acts as an internal engineering checkpoint. To proceed, the selection must be formally justified, ensuring no licensing or infrastructure constraints are violated. Table 6 summarises the governance gates and required evidence.

**Table 6.** Governance gates and required artefacts for Base AI Model.

Governance gate	Required Evidence/Artefact
The model selection is justified based on sourcing paths, licensing, and operational constraints.	Documented base-model selection rationale.
The selected model (and any adapted variant) is fully traceable.	Versioned reference to the selected base model in a registry.
Potential gaps, bias risks, and constraints are identified for downstream testing.	Initial limitations and assumptions statement to be tested during validation.
Final Release Approval.	Versioned and registered model reference(s) suitable for controlled reuse (Model Card ^44^).

**Primary responsibility.** The Provider leads this component, as they maintain the model registries and hold the technical expertise to adapt the architecture. However, they must strictly operate within the licensing and infrastructure constraints established by the Deployer.

***Data Processing & Governance***

**Challenge.** In security contexts, Developers often face strict operational restrictions preventing the use of raw operational data. Consequently, they must rely on synthetic, simulated, or proxy datasets [2]. If these datasets are ingested without strict governance, provenance tracking, and privacy controls, it severely compromises the credibility of downstream validation and introduces unacceptable operational risks.

**Objective.** This component operationalises the data governance strategy defined in the Definition & Design stage. It provides that all data used for development complies with sourcing constraints, privacy and access requirements, traceability expectations, and documented assumptions, so that the resulting dataset can be audited and reused under controlled conditions.

**Figure 6.**
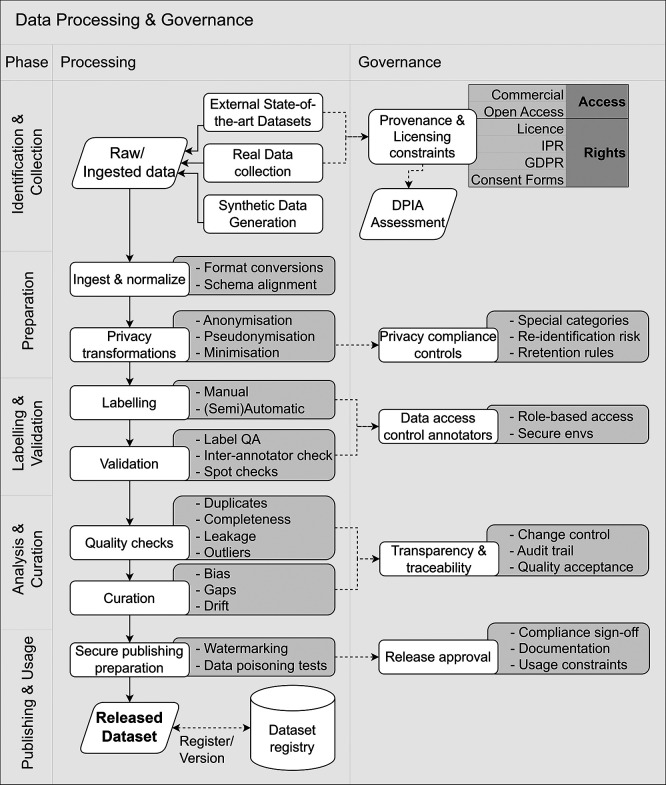
Data Processing & Governance pipeline with embedded lifecycle controls.

**Operational Approach.** Figure 6 illustrates how the Data Processing & Governance is treated as a staged pipeline in which each processing step is paired with corresponding governance controls, culminating in a dataset release that is registered and versioned.

The workflow proceeds through sequential phases, with governance obligations integrated as controls and checkpoints rather than applied retrospectively:

1. **Identification & collection**. Data sources are identified (e.g., external datasets, operationally collected data where permitted, or synthetic/simulated data). For each source, provenance and licensing constraints are recorded, and where applicable, privacy/legal feasibility is assessed (including DPIA-triggering conditions).
2. **Preparation**. Raw/ingested data is normalised and aligned (format conversion, schema harmonisation), and privacy-preserving transformations are applied when needed (e.g., anonymisation, pseudonymisation, minimisation).
3. **Labelling & validation**. Where annotations are required, labelling workflows are defined, and quality is assessed (e.g., spot checks, inter-annotator agreement), alongside enforcement of role-based access controls for annotators and secure working environments.
4. **Analysis & curation**. Dataset quality is assessed (e.g., duplicates, leakage, completeness, outliers), and curation activities address representativeness risks (bias, gaps, potential drift relative to the intended context of use). Traceability artefacts are updated to preserve an auditable history of changes.
5. **Publishing & usage**. Before use in training, a release approval checkpoint records usage constraints, documentation status, and (where relevant) security considerations. The outcome is a released dataset suitable for controlled reuse.

**Key activities**

• Register datasets and maintain provenance and licensing records from acquisition to release.
• Implement access control consistent with provider/deployer roles and permitted handling conditions.
• Document dataset composition, preprocessing, and all transformations applied across pipeline phases.
• Apply privacy-preserving controls and data minimisation measures where required by the data and context.
• Validate labels and dataset quality, and document representativeness assumptions and limitations.
• Establish a dataset release decision with versioning to support traceability and reproducibility.

Where synthetic, simulated, or proxy datasets are used due to operational restrictions, their representativeness hypotheses and boundary conditions must be explicitly documented, as they directly affect risk assumptions and downstream validation credibility.

**Governance Gate and Evidence.** Before the data can be utilised for Model Training, it must pass a release approval checkpoint ( Table 7). Furthermore, where synthetic, simulated, or proxy datasets are used, their representativeness hypotheses and boundary conditions must be explicitly documented, as they directly affect risk assumptions.

**Table 7.** Governance gates and required artefacts for Data Processing & Governance.

Governance Gate	Required Evidence/Artefact
The dataset is fully traceable, and rights/access constraints are established.	Provenance/licensing record and access-control description (including usage constraints).
Data processing steps (privacy, normalisation, quality control) are verified.	Dataset documentation describing sourcing, composition, preprocessing, and quality controls.
Representativeness hypotheses, boundary conditions, and gaps are acknowledged.	Dataset limitations statement, including representativeness assumptions and known gaps.
Final Release Approval.	Versioned and registered dataset reference(s) suitable for controlled reuse (Datasheet ^45^).

**Primary responsibility.** The Provider executes the technical data pipeline (normalisation, curation, versioning). However, the Deployer is responsible for defining the privacy constraints, approving the DPIA-triggering conditions, and enforcing access control rules, particularly if any operational data is involved.

***Model Training***

**Challenge.** Model training in security-oriented AI projects faces two main challenges. First, even after the data preparation stage, the available dataset may remain limited due to operational restrictions, privacy constraints, or the scarcity of representative events. Training strategies must therefore be adapted to the available data volume while still achieving acceptable performance and robustness. Second, the training process must maintain strict traceability and configuration control. Different training runs may involve variations in hyperparameters, architectures, optimisation strategies, or dataset versions, and without proper documentation, these differences can compromise reproducibility, validation, and later auditability.

**Objective.** The objective of the Model Training component is to produce a trained model that satisfies the predefined validation criteria while ensuring full traceability of the training process. This involves combining the selected base model with the released, versioned dataset within a controlled training workflow that records model configurations, training parameters, and dataset references. Unlike exploratory research environments, evaluation metrics and acceptance thresholds are defined before training, ensuring that the resulting model remains aligned with the intended purpose, system boundaries, and governance requirements established during the Definition & Design stage.

**Figure 7.**
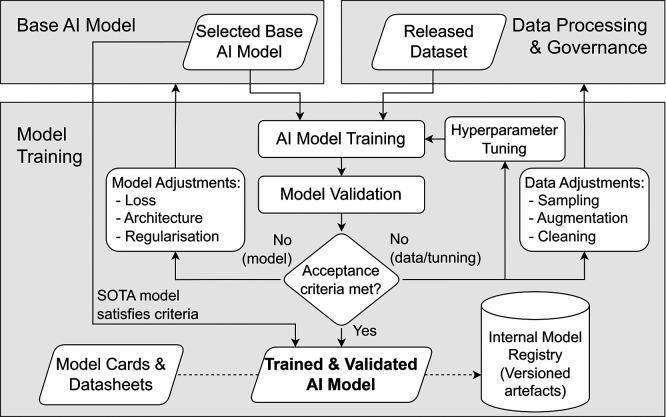
Controlled model training and validation loop.

**Operational Approach.** As illustrated in Figure 7, model training is treated as an iterative but controlled process in which the selected base model and the released, versioned dataset feed into a structured training and validation loop [3]. If the predefined acceptance criteria are not met, controlled iterations may adjust the model configuration, hyperparameters, or dataset preparation (e.g., sampling, augmentation, or cleaning). Each iteration must remain bounded by the intended purpose, predefined metrics, and configuration-control requirements established during the Definition & Design stage.

Where an existing state-of-the-art model already satisfies the predefined acceptance criteria, the training loop may be bypassed, and the model can be directly adopted, provided that its provenance, licensing conditions, and limitations are documented. All resulting artefacts are versioned in the internal model registry, and the stage outputs a trained and internally validated model together with the associated documentation artefacts.

**Key activities**

• Configure training parameters consistent with the predefined validation plan.
• Execute model training on the formally released and versioned dataset.
• Perform model validation against predefined performance and robustness criteria.
• Identify issues and execute structured iterations involving model-side adjustments (e.g., architecture, regularisation), hyperparameter tuning, or data-side adjustments (e.g., augmentation policies) under strict configuration control.
• Document the performance gap, including representativeness limitations and known domain shifts.

**Governance Gates and Evidence.** Although model training is primarily an engineering activity, it remains constrained by governance artefacts. The model cannot be passed to the Software System Integration phase unless its iterations align with the original intended purpose and predefined metrics. Table 8 describes the governance gates for the Model Training step.

**Table 8.** Governance gates and required artefacts for Model Training.

Governance Gate	Required Evidence/Artefact
The model has met predefined performance and robustness criteria without scope drift.	Model validation report.
Training iterations are fully reproducible.	Training configuration and hyperparameter record.
The “data gap” (domain shifts, representativeness limits) is explicitly acknowledged for downstream evaluation.	Performance summary and documented limitations.
Final Release for Integration.	Versioned and internally validated trained model, alongside updated model and dataset documentation artefacts (e.g., Model Cards ^44^ and Datasheets for Datasets ^45^).

**Primary responsibility.** The Model Training component is primarily led by the Provider. However, the Deployer may participate in controlled evaluation environments or structured review checkpoints, particularly where operational constraints require on-premises testing or restricted data handling.

***Software System Integration***

**Challenge.** Operational AI systems depend on multiple runtime components and third-party libraries. In restricted environments, undocumented dependencies, licensing conflicts, or unpatched vulnerabilities pose severe operational, security, and legal risks. Furthermore, embedding a standalone model into a broader application stack carries the risk of inadvertently altering its intended purpose or validated behaviour without a structured review.

**Objective.** The objective is to translate the internally validated AI model into an operational AI-enabled software solution suitable for controlled deployment. It must integrate the trained model into a broader software architecture that satisfies the functional and non-functional requirements defined during the Definition & Design stage, while strictly preserving traceability, reproducibility, and compliance with governance constraints.

**Figure 8.**
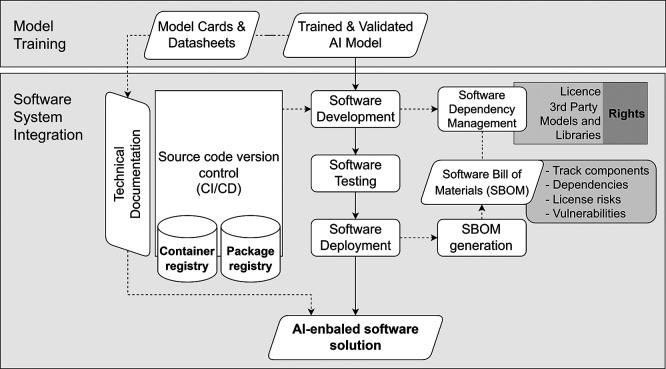
Software system integration with dependency management and licensing controls.

**Operational Approach.** As illustrated in Figure 8, the validated model is embedded into a deployable system through structured integration, testing, and controlled release practices. This involves integrating the AI component with application programming interfaces (APIs), business logic, data connectors, user interfaces, and monitoring subsystems. To manage dependency complexity and provide transparency, teams adopt a software supply-chain approach, utilising a Software Bill of Materials (SBOM) to track provenance, assess vulnerabilities, and review license compliance. Where deployments rely on specialised hardware (e.g., accelerated compute platforms), compatibility between hardware, drivers, toolchains, and runtime components is meticulously validated.

**Key activities**

• Integrate the model into the broader architecture, including APIs, rule enforcement, and logging subsystems.
• Version, test, and document all dependencies, including ML runtimes, inference libraries, and supporting frameworks to ensure debuggability.
• Assess all third-party components (including external models) for compliance with applicable licensing and rights constraints.
• Conduct structured testing practices, including unit and integration testing, system-level functional testing, and performance/stress testing on inference pathways.
• Generate an SBOM to support provenance tracking, vulnerability assessment, and reproducible builds.
• Compile an extended technical documentation package covering security controls, monitoring configurations, and operational rollback mechanisms.

**Governance Gate and Evidence.** Software implementation remains constrained by the artefacts established in earlier stages. Integration must not alter the intended purpose, system boundaries, or validated model behaviour. Significant architectural changes may require re-evaluation under the Definition & Design gate. Table 9 shows the governance gates and required evidence in this step.

**Table 9.** Governance gates and required artefacts for Software Integration System.

Governance Gate	Required Evidence/Artefact
Dependency complexity, vulnerabilities, and licensing constraints are fully mapped.	Recorded dependency and component inventory (SBOM).
The system meets functional requirements without altering validated model behaviour.	Integration and test report.
Deployment procedures are highly reproducible and reversible.	Build and deployment specification (including rollback mechanisms).
The system is fully documented to support compliance and operational handover.	Extended technical documentation package.
Final Release for Validation.	Deployable AI-enabled software artefact (versioned).

**Primary responsibility.** This component is primarily Provider-led. However, Deployers may participate in staging infrastructure verification and operational readiness reviews, particularly where deployments occur within controlled or restricted institutional environments.

### Validation

**Challenge.** In many security and law-enforcement deployments, operational data cannot be shared with development teams due to legal, privacy, or operational security constraints. Therefore, the main challenge resides in validating the solution with real data or in an operational environment. This step of the methodology recommends validation patterns that protect operational data while still producing auditable evidence.

**Objective.** Validation is the process of verifying that the AI-enabled software solution performs as intended in its operational environment. This stage occurs after the system is deployed on the user’s premises, where it is exercised under real-world conditions to ensure that it meets both functional and non-functional acceptance criteria, as defined during the Definition & Design phase.

**Figure 9.**
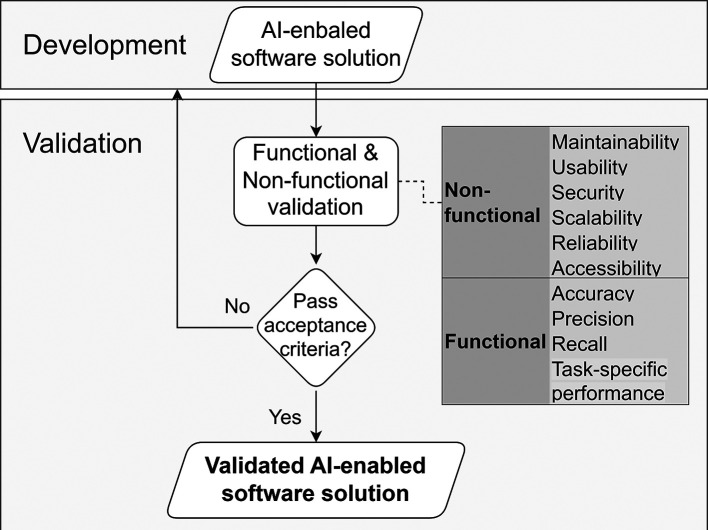
Validation of the AI-enabled software solution with functional and non-functional acceptance criteria.

**Operational Approach.** As illustrated in Figure 9, validation relies on previously unseen, representative operational data (or a clearly defined hold-out sample) to evaluate system performance in realistic conditions, ensuring results reflect actual deployment scenarios rather than development-time tuning. It combines structured user feedback (surveys, incident reporting) with objective telemetry (model inputs, predictions, timestamps, overrides) to assess operational performance.

To navigate data constraints, the methodology recommends three specific validation patterns. These patterns operate at different levels: the first defines the execution setting, the second defines the acceptance mechanism, and the third defines how validation evidence is partitioned and preserved for auditability:

• **Bring a solution to the data evaluation.** A validation **execution pattern** in which the Provider’s solution is transferred to, and evaluated within, the Deployer’s secure environment, so that operational data remains under the Deployer’s control and is never disclosed externally. The Provider receives only aggregated evaluation outputs, such as summary metrics and error analyses.
• **On-premises gated acceptance testing.** A validation **governance pattern** in which the Deployer assesses the solution within its own infrastructure against predefined acceptance criteria, decision thresholds, and testing conditions established during the Definition & Design stage. The process concludes with a formal pass/fail decision, documented sign-off, and explicit allocation of stakeholder responsibilities.
• **Provider–deployer evidence split.** A cross-cutting evidence management pattern in which validation artefacts are divided between Provider and Deployer according to their respective roles and access rights. The Provider contributes development-side evidence (e.g., model documentation, training records, validation protocol, version identifiers), while the Deployer contributes operational evidence (e.g., test execution records, results on operational data, acceptance decisions, and monitoring configurations). Together, these artefacts form a joint validation evidence pack without requiring disclosure of raw operational data.

These patterns support that proxy or synthetic datasets remain useful for pre-validation and engineering iteration, while final validation is grounded in controlled evaluation on operational data with traceable, role-separated evidence.

**Key activities**

• Agree on measurable acceptance criteria before validation begins, including performance metrics (accuracy, precision, recall) and task-specific measures (latency, false-alarm rates).
• Assess key system qualities through robustness testing (distribution shifts), security assessment (threat modelling, model extraction), safety evaluation (false positive/negative consequences), and explainability verification.
• Verify non-functional acceptance criteria, including maintainability, usability, scalability, reliability, security validation (SBOM, licensing), and accessibility.
• Execute acceptance testing using predefined metrics, minimum sample-size rules, and statistical decision thresholds.

**Governance Gate and Evidence.** Validation deliverables ( Table 10) must be documented and preserved for traceability and auditability. A formal sign-off assigns responsibilities and records pass/fail outcomes together with supporting evidence. If any gate fails, remediation actions must be documented, with timelines and measurable exit criteria established before the next validation round.

**Table 10.** Governance gates and required artefacts for Validation.

Governance Gate	Required Evidence/Artefact
The system passes operational evaluation on unseen data without exposing raw data to the provider.	Validation dataset specification and snapshots.
Functional and non-functional criteria are met according to statistical thresholds.	Test results, logs, and completed user feedback surveys.
System limits, cyber vulnerabilities, and failure modes are identified and mitigated.	Summaries of robustness and security testing.
The system is fully documented and prepared for operational handover.	Operational runbooks, maintenance SLAs, and training materials for end users.
Final Operational Acceptance.	Signed acceptance records, alongside Model Card and dataset registry updates reflecting the validated version.

**Primary Responsibility.** The Deployer takes the lead execution role, conducting the acceptance testing entirely within their infrastructure. The Provider contributes design and development evidence (model documentation, training procedures, validation protocols), resulting in a joint evidence pack that supports auditability without transferring raw operational data.

### Monitoring

**Challenge.** Models deployed in dynamic operational environments are susceptible to data drift, emerging security vulnerabilities, and evolving legal landscapes. Point-in-time audits are insufficient for systems that change frequently. Without continuous monitoring, a once-compliant AI system can quickly become a legal or operational liability.

**Objective.** Post-deployment monitoring ensures that the AI-enabled solution continues to meet **functional** and **non-functional** requirements over time. It combines **continuous telemetry** and **alerting** systems with **periodic reviews** to support ongoing compliance and operational performance. As a continuous process with feedback loops, it feeds into system re-validation and corrective actions when needed.

**Figure 10.**
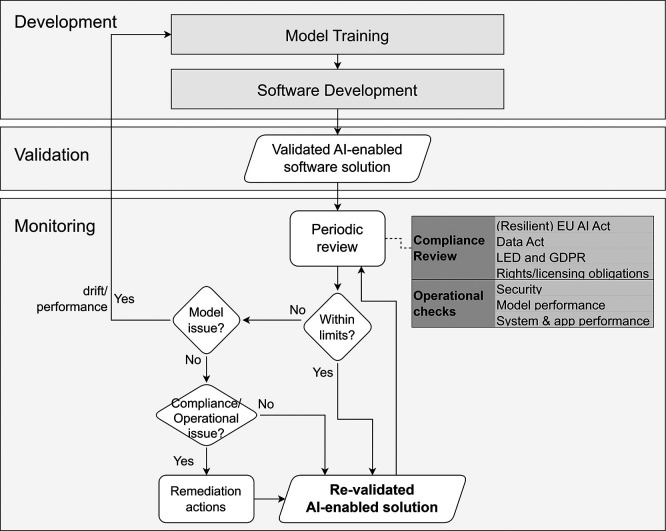
Monitoring and periodic review process for AI-enabled solution validation.

**Operational Approach.** As shown in Figure 10, monitoring relies on evidence from two key streams that feed into periodic reviews: **Compliance reviews** (verifying alignment with legal, ethical, and licensing obligations) and **Operational checks** (tracking security, performance, and stability). If the system remains within predefined limits, it is re-validated for the current period. If it deviates from acceptable thresholds, a root cause assessment determines the corrective action. Model-related issues (e.g., performance drift) trigger a return to the Development stage for retraining or redesign. Conversely, operational or compliance-related issues (e.g., security vulnerabilities) lead to direct remediation efforts without returning to development.

**Key activities**

• Conduct compliance reviews to support the system operates within the scope of the EU AI Act, GDPR, Data Act, and Law Enforcement Directive (LED).
• Verify ongoing adherence to rights and licensing constraints for third-party components (models, libraries, external datasets).
• Execute security monitoring, including SBOM-driven vulnerability scanning, detecting suspicious access patterns, and running incident response and rollback procedures.
• Track model performance metrics over time to detect drift and data quality degradation, using controlled retraining triggers (e.g., sustained metric drops).
• Monitor system and application performance for unexpected behaviour or failures.
• Document all corrective actions, escalation procedures, and significant updates to check that the solution remains auditable and accountable.

**Governance Gate and Evidence.** All outcomes from the monitoring process must be thoroughly documented for traceability. Table 11 summarises these governance gates and requirements.

**Table 11.** Governance gates and required artefacts for Monitoring.

Governance Gate	Required Evidence/Artefact
The system remains aligned with legal, ethical, and licensing obligations.	Compliance review records.
Security, stability, and model performance limits are maintained.	Monitoring telemetry, logs, and performance/security monitoring reports.
Deviations are resolved via appropriate remediation or retraining.	Corrective action records and approvals.
Periodic Re-validation.	Updated Model Card and dataset/version registry.

**Primary responsibility.** This phase is a joint effort between the Deployer (operations) and the Provider (support). The Deployer oversees the operational telemetry, security monitoring, and compliance within their restricted infrastructure. The Provider assists with root-cause analysis, software patching, and managing the retraining process if model drift triggers a return to the development stage.

## Cross-cutting requirement: systematic documentation and traceability across lifecycle stages

A fundamental cross-cutting requirement of the proposed security-oriented AI lifecycle is the systematic documentation of all activities performed within each of the five principal stages. This documentation is not a secondary reporting exercise but a core engineering and governance mechanism that enables traceability, accountability, and operational trustworthiness.

For each stage, all key decisions, processes, configurations, and outcomes must be recorded through structured and versioned artefacts (e.g., decision logs, risk assessments, dataset registries, model documentation, validation reports, and monitoring records). This ensures that the full **lifecycle of the AI system can be reconstructed**, audited, and justified at any point in time, in line with EU regulatory expectations regarding record-keeping, transparency, and accountability.

Beyond compliance, systematic documentation plays a critical role in bridging the gap between development and operational deployment. It provides the foundation for transforming a research prototype into a commercial, operational-grade solution, enabling maintainability, reproducibility, and controlled evolution of the system over time. In particular, it supports **knowledge transfer between Providers and Deployers**, facilitates certification or conformity assessment processes, and reduces operational risks associated with personnel turnover or system updates.

From an end-user perspective, well-structured documentation contributes directly to **acceptability and trust**. By making system behaviour, limitations, and decision logic explicit, it enables users to understand the capabilities and boundaries of the AI tool, fostering informed and conscious use rather than blind reliance. This is especially critical in security and law-enforcement contexts, where decisions may have significant legal and societal consequences.

Finally, documenting lifecycle activities ensures clear accountability allocation between stakeholders. By explicitly recording who performed which action, under which assumptions, and with what evidence, the framework supports responsibility tracking across organisational boundaries, which is essential in multi-actor EU security ecosystems.

In this methodology, **documentation is therefore treated as a first-class artefact of the lifecycle**, embedded at each governance gate and continuously maintained, rather than produced retrospectively.

## Compliance mapping: from EU obligations to lifecycle artefacts

To operationalise EU regulatory requirements within engineering workflows, we map key obligations under the EU AI Act and the EU data protection framework (GDPR/LED) onto lifecycle stages, accountable roles, and concrete evidence artefacts. The objective is not to restate legal provisions, but to translate them into auditable engineering outputs and decision checkpoints that can be produced and verified throughout the lifecycle.

Table 12 summarises this mapping by linking each obligation cluster to:

(i) the relevant lifecycle stage(s);
(ii) the primary responsible actor(s), distinguishing provider and deployer responsibilities; and
(iii) the categories of evidence artefacts that can demonstrate compliance in practice.

**Table 12.** Core regulatory obligations mapped to lifecycle stages, actors, engineering artefacts and produced evidence.

Regulatory Obligation cluster (EU)	Lifecycle stage(s)	Primary actor	Evidence artefacts (categories)
Risk management and change control (AI Act, high-risk)	Definition & Design; Development; Validation; Monitoring	Provider and Deployer	Risk register; mitigation plan; residual risk justification; change-impact records; release signoffs
Data governance and data quality (AI Act; GDPR/LED principles)	Definition & Design; Development	Provider (with controller constraints)	Data sourcing/provenance record; dataset description; quality/representativeness summary; lawful-use and reuse constraints
Technical documentation and record-keeping (AI Act; GDPR/LED accountability)	Development; Deployment; Monitoring	Provider (design) and Deployer (operation)	Technical documentation pack; traceability overview; logging requirements; audit trail policy (access/retention at conceptual level)
Transparency and user information (AI Act; GDPR fairness/notice where applicable)	Validation; Deployment	Provider and Deployer	User information pack; limitations/appropriate-use statement; performance/uncertainty communication summary
Human oversight (AI Act)	Definition & Design; Validation; Monitoring	Provider (design) and Deployer (implementation)	Oversight concept; roles/escalation procedures; intervention policy; oversight effectiveness review records
Accuracy, robustness and cybersecurity (AI Act)	Development; Validation; Monitoring	Provider and Deployer	Validation protocol; robustness evaluation summary; security assurance summary; known failure modes and mitigations
Post-market monitoring and incident handling (AI Act post-market monitoring)	Monitoring	Deployer (ops) and Provider (support)	Monitoring plan; incident workflow; monitoring reports; corrective action records; re-validation triggers

The selected clusters reflect core AI Act requirements for high-risk systems, such as a risk management system, data governance, technical documentation and record-keeping, transparency and user information, human oversight, accuracy/robustness/cybersecurity, and post-deployment monitoring; together with GDPR/LED accountability expectations where personal data is processed.

This mapping, specified in Table 12, enables compliance to function as an engineering constraint rather than a post hoc documentation exercise. It is intentionally conceptual and tool-agnostic: it specifies artefact categories and governance checkpoints (e.g., risk register updates, dataset provenance records, validation reports, monitoring reviews, release signoffs) that can be implemented using different tools depending on project context and infrastructure constraints, including restricted or isolated environments.

In projects with broader public-facing implications, ethics and fundamental-rights review may need to be complemented by a more explicit societal-impact assessment, depending on the nature of the use case and the institutional governance context.

The table also clarifies the provider–deployer split: Providers typically produce the system-level evidence pack associated with design choices, training and validation, and traceability-by-design, while deployers operationalise context-dependent controls such as oversight procedures, logging and monitoring, incident handling, and any deployment-specific impact assessments. Across projects, the same evidence categories may be implemented differently (e.g., different registries, logging stacks, or security tooling), without changing the underlying compliance logic captured in the mapping.

## Discussion and limitations

This paper has proposed an EU-centric lifecycle methodology for developing AI systems in security and law enforcement contexts. The need for this integrated approach arises from three converging realities: (1) strict data-access constraints prevent the use of raw operational data for model training; (2) EU regulation imposes substantive lifecycle obligations on high-risk systems, including risk management, data governance, and human oversight; and (3) standard MLOps practices still lack widely adopted mechanisms for continuous legal and ethical compliance. Together, these factors make a unified, auditable, and operationally oriented methodology necessary for AI trustworthy deployment.

### Practical implications and continuous assurance

The presented methodology addresses these gaps by embedding concrete controls throughout five developmental stages: Matchmaking, Definition & Design, Development, Validation, and Monitoring. Rather than treating compliance as a post hoc checklist, this framework maps regulatory duties directly to engineering artefacts.

Two primary practical implications emerge from this approach:

• **Compliance must be continuous, embedded at the appropriate points in the lifecycle, and machine-readable **: Point-in-time audits are insufficient for systems subject to frequent updates, configuration changes, and data drift. Instead, compliance checks should be integrated into the lifecycle at predefined governance gates and engineering checkpoints, ensuring that regulatory and accountability requirements are verified when relevant decisions are made. Embedding these checks within development pipelines, through automated and artefact-driven mechanisms, materially reduces the cost and latency of regulatory evidence collection. Engineering practices such as generating Software Bills of Materials (SBOMs), maintaining Model Cards and Datasheets, and automating vulnerability and licence checks support reproducibility, traceability, and software supply-chain assurance.
• **Legal scoping and licensing are foundational design constraints**: Traditionally, compliance checks during development focused primarily on Intellectual Property Rights (IPR) and software licensing associated with third-party models and libraries. While these aspects remain essential, since strong copyleft or restrictive licences may conflict with the confidentiality requirements of law-enforcement deployments, today they must be complemented by regulatory compliance considerations. In particular, the intended deployment context must be assessed against obligations arising from the GDPR, potential DPIA requirements, and the EU AI Act risk classification framework. Consequently, legal scoping now extends beyond licence compatibility to include early verification and monitoring that the system’s intended use can be lawfully deployed within the applicable regulatory environment.

A further practical implication concerns ethical governance. In the proposed lifecycle, ethics review is not treated as a peripheral consultation but as a potential go/no-go gate. Depending on the project context, ethics committees or equivalent oversight bodies within the participating organisations may require redesign, impose conditions, or block progression altogether. This is particularly important in security-oriented projects, where even technically feasible systems may be unacceptable because of proportionality, fairness, or fundamental rights concerns.

### The “Synthetic Data Trap” and validation mitigations

A profound operational challenge inherent to security AI development is the “synthetic data trap”. Because LEAs cannot share operational data for iterative training, developers lean heavily on synthetic samples, public benchmarks, or generative AI. This reliance often glosses over the massive domain shift between lab-environment training data and real-world operational realities. Expecting models trained on generic data to perform reliably in high-stakes operational scenarios introduces severe operational risk.

The methodology mitigates this risk by fundamentally restructuring the validation phase. As illustrated in *Illustrative application of the lifecycle methodology,* ^8^ the inability to access ground truth data during development is countered by the “Bring-solution-to-data” validation pattern. By delivering the AI-based system solution to the LEA, acceptance testing is conducted entirely within the deployer’s secure infrastructure using real operational hold-out data. This supports that final performance is measured against actual operational realities without violating data export restrictions, yielding role-separated, auditable evidence.

### Limitations and future work

A limitation of the present framework is that it does not include a standalone societal-impact lane as a mandatory component for every project. Social issues have been considered within the ethical and governance assessment, particularly through proportionality analysis, human oversight, and committee-based review. However, not all technological developments within security research affect society directly or to the same degree, and for that reason, societal assessment was not formalised as a separate compulsory track in the core lifecycle. Nevertheless, in projects with broader public-facing effects, such as biometric surveillance, or systems likely to shape citizen rights or public-space behaviour, a more explicit societal-impact assessment would be necessary and should be incorporated into the governance gate.

The primary limitation of this work is that it presents a methodology rather than a widespread empirical evaluation. While grounded in practitioner literature, EU regulatory texts, European projects like STARLIGHT [4], LAGO [5], AP4AI [6] and MultiRATE [7], and other European initiatives like ALTAI [8], broader adoption will require significant organisational-level change and new tooling. Furthermore, the EU regulatory landscape, including interactions between the AI Act, the LED, and national laws, is still evolving, meaning continuous legal monitoring is required for practical implementation.

Future work must focus on bridging the remaining data gaps during the development phase itself. Because bringing the model to the data is currently a validation-stage solution, exploring provider–deployer evidence split, embedding technical experts within LEA premises, or providing developers with highly detailed statistical descriptions of operational data remain open challenges. Additionally, research should prioritise prototyping automated audit hooks for popular MLOps stacks, publishing exemplar artefacts (e.g., context-specific Model Cards and SBOMs), and running further field pilots with LEA partners to refine acceptance thresholds.

In conclusion, deploying AI-based systems safely in security contexts requires marrying rigorous engineering controls with embedded legal and ethical governance. By making compliance auditable, reproducible, and native to the MLOps lifecycle, this methodology reduces deployment risks and increases the trustworthiness of AI systems used by law enforcement organisations across the EU.

## Ethics and consent

Ethical approval and consent were not required.

## Disclaimer

The views and opinions expressed in this paper are those of the authors and do not necessarily reflect the official policy or position of any affiliated institutions, agencies, or organisations. While every effort has been made to ensure the accuracy of the information presented, the authors cannot be held responsible for any errors, omissions, or consequences arising from the use of this content. This paper is intended for academic purposes and is not a substitute for legal, technical, or professional advice. All third-party sources, tools, and frameworks mentioned in this paper are used under the terms of their respective licenses.

## Data Availability

The operational data used for validation in the STARLIGHT worked example consists of CCTV-based continuous crowd-monitoring video streams. Due to the presence of personally identifiable information and strict operational security policies governed by the Law Enforcement Directive (LED) and the General Data Protection Regulation (GDPR), this data is highly sensitive and remains strictly within the premises of the participating Law Enforcement Agencies (LEAs). The restrictions on this data are therefore:
• Legal restrictions: personal data processing under GDPR/LED frameworks • Ethical restrictions: use governed by Data Protection Impact Assessments (DPIAs) and institutional ethical review procedures • Security restrictions: data cannot leave controlled LEA environments (including restricted or air-gapped infrastructures) Access to the data is not publicly available. Requests for access must be submitted through the appropriate institutional channels and will be assessed by the data-controlling LEAs on a case-by-case basis, subject to legal, ethical, and security approval. Initial contact regarding data access requests can be made via the corresponding author ( maramburu@vicomtech.org) or via the project coordination contact ( starlight@cea.fr), who will redirect the request to the relevant data-controlling authority. Access, if granted, is subject to formal agreements, compliance with GDPR/LED requirements, and, where applicable, institutional affiliation and security clearance. No underlying datasets are publicly available for this study. The validation described in the manuscript relies on operational law-enforcement data that cannot be shared due to legal, ethical, and security constraints, as detailed above. No raw or derived datasets can be made accessible outside the controlled environments of the participating LEAs. Toolkit for EU-Centric Law Enforcement AI Lifecycle. Available at: 10.5281/zenodo.19482658, https://zenodo.org/records/19482658. ^8^ DOI: 10.5281/zenodo.19482658 This repository contains the following underlying data:
• FCT-Specific Model Card Template. • FCT-Specific Datasheet Template. • “Bring-Solution-to-Data” Validation Protocol and Acceptance Report. • Legal & Ethical Pre-Assessment Matrix (adapted from AP4AI/HLEG). • DevSecOps and MLOps tooling matrix for FCT Research AI Projects. • AI SBOM template for License and Vulnerability Tracking. • Illustrative application of the lifecycle methodology (STARLIGHT use case). All materials are openly available and do not require login or embargo. Data are available under the terms of the Creative Commons Attribution 4.0 International license (CC-BY 4.0). https://creativecommons.org/licenses/by/4.0/legalcode As no raw operational data can be shared, the extended data repository serves as the primary reproducibility resource for this study.

## Footnotes

[1] Social issues have been considered, but as not all the technological developments directly affect society, it has been decided to not integrate it. However, it would be necessary in some cases.

[2] A substitute dataset used in statistical analysis or machine learning when the actual data is unavailable or difficult to obtain. It helps researchers or models make inferences about the target variable by providing a related but more accessible alternative.

[3] Other training and validation paradigms, such as federated training, can also be applied. However, for the purposes of this methodology, they are considered to face the same core challenges addressed in this section, particularly with respect to data constraints, traceability, validation, and governance.

[4]
https://starlight-h2020.eu/

[5]
https://lago-europe.eu/

[6]
https://ap4ai.eu/

[7]
https://www.multirate.eu/

[8]
https://altai.insight-centre.org/
