# Supplementary material for: From prototype to deployment: An EU-centric lifecycle framework for law enforcement AI
Source: Open Res Eur. 2026 Apr 30;6:141. [Version 1] doi: 10.12688/openreseurope.21859.1 (PMC13231134; doi:10.12688/openreseurope.21859.1)
Supplement: Peer review history

# Reviewer report: Reviewer response for version 1

**DOI:** 10.21956/openreseurope.23646.r73556

**Published:** 2026-06-02

**Authors:** Giovanni Sartor

**Affiliations:** 1 University of Bologna, Bologna, Italy

Overview:

 The paper presents a detailed and timely contribution addressing the development and deployment of AI systems in security-sensitive and law-enforcement contexts within the EU regulatory framework. The authors propose a comprehensive lifecycle methodology structured around five stages  Matchmaking, Definition & Design, Development, Validation, and Monitoring  aimed at integrating legal, ethical, and technical requirements throughout the entire AI development process. The work is particularly valuable for its effort to bridge the gap between regulatory obligations (e.g., AI Act, GDPR, LED) and engineering practices, providing a structured overview of the concrete steps involved in developing trustworthy AI systems for high-risk domains.

 Strengths

 One of the main strengths of the paper is its high level of detail. The proposed framework is carefully articulated, and each phase is supported by governance gates, documentation requirements, and operational considerations. The discussion on validation under restricted-data conditions and the integration of compliance requirements into engineering workflows are highly relevant.

 Weaknesses:

 However, the contribution remains largely theoretical. While the framework is well described conceptually, the paper would benefit from a more concrete and operational demonstration of how the methodology can be implemented in practice. Although the work is positioned specifically within the law-enforcement domain, many aspects of the proposed lifecycle appear to be generic and potentially applicable to any high-risk AI system under the EU AI Act. The framework currently lacks elements that clearly distinguish it as uniquely tailored to the law-enforcement context beyond the general discussion of restricted operational data and regulatory constraints.

 Recommendations:

 The paper would benefit from a more balanced integration between the methodological description and the practical application of the framework. At present, the methodology is explained in considerable detail, while the STARLIGHT use case is only briefly mentioned and mainly described elsewhere. It may therefore be beneficial to reduce some of the methodological exposition and incorporate a more concrete presentation of the STARLIGHT scenario directly within the paper. This would strengthen the practical dimension of the contribution, better illustrate how the framework operates in a realistic law-enforcement setting, and potentially reveal additional operational challenges, overlooked interactions, or domain-specific requirements that are not fully visible in the current theoretical presentation.

Is the rationale for developing the new method (or application) clearly explained?

Yes

Is the description of the method technically sound?

Yes

Are the conclusions about the method and its performance adequately supported by the findings presented in the article?

Yes

If any results are presented, are all the source data underlying the results available to ensure full reproducibility?

No source data required

Are sufficient details provided to allow replication of the method development and its use by others?

Yes

Reviewer Expertise:

Law, artificial intelligence, legal theory, ICT law

I confirm that I have read this submission and believe that I have an appropriate level of expertise to confirm that it is of an acceptable scientific standard.

# Reviewer report: Reviewer response for version 1

**DOI:** 10.21956/openreseurope.23646.r74140

**Published:** 2026-05-14

**Authors:** Stephanie Ness

**Affiliations:** 1 University of Vienna, Vienna, Austria

The authors explain why ordinary MLOps methods do not fit all law enforcement settings. They also explain why GDPR, the Law Enforcement Directive, and the EU AI Act create duties that must be tracked during Matchmaking, Definition and Design, Development, Validation, and Monitoring.

 The method is partly technically sound. The structure is clear and the terms are correctly used. There is a role split between provider and deployer.

 The main weakness is that some steps are still described at the method level only. The validation stage would benefit from more technical detail. The authors should say how acceptance thresholds are chosen. This is needed because many law enforcement tasks involve rare events, in which accuracy alone can be misleading.

 Equally, the method is partly replicable. A reader can follow the five stages, but may not be able to apply the method in the same way. The authors should add at least two concrete examples and distinguish those.

 No source data is required for full reproducibility in the usual sense. This is a method article, not an empirical model comparison. The operational data in the example cannot be shared, which is reasonable in this setting. The “bring-solution-to-data” pattern is a suitable approach for testing the system without moving sensitive police data.

Is the rationale for developing the new method (or application) clearly explained?

Yes

Is the description of the method technically sound?

Yes

Are the conclusions about the method and its performance adequately supported by the findings presented in the article?

Yes

If any results are presented, are all the source data underlying the results available to ensure full reproducibility?

No source data required

Are sufficient details provided to allow replication of the method development and its use by others?

Partly

Reviewer Expertise:

Cybersecurity, AI governance, EU digital regulation, data protection, and secure AI systems.

I confirm that I have read this submission and believe that I have an appropriate level of expertise to confirm that it is of an acceptable scientific standard.
